# Comparative Evaluation of Heterologous Production Systems for Recombinant Pulmonary Surfactant Protein D

**DOI:** 10.3389/fimmu.2014.00623

**Published:** 2014-12-08

**Authors:** Daniela Salgado, Rainer Fischer, Stefan Schillberg, Richard M. Twyman, Stefan Rasche

**Affiliations:** ^1^Fraunhofer Institute for Molecular Biology and Applied Ecology IME, Aachen, Germany; ^2^Institute for Molecular Biotechnology, RWTH Aachen University, Aachen, Germany; ^3^TRM Ltd., York, UK

**Keywords:** biopharmaceuticals, heterologous production platform, pulmonary surfactant, recombinant protein yield, recombinant surfactant protein D, respiratory distress syndrome

## Abstract

Commercial surfactant products derived from animal lungs are used for the treatment of respiratory diseases in premature neonates. These products contain lipids and the hydrophobic surfactant proteins B and C, which help to lower the surface tension in the lungs. Surfactant products are less effective when pulmonary diseases involve inflammatory complications because two hydrophilic surfactant proteins (A and D) are lost during the extraction process, yet surfactant protein D (SP-D) is a component of the innate immune system that helps to reduce lung inflammation. The performance of surfactant products could, therefore, be improved by supplementing them with an additional source of SP-D. Recombinant SP-D (rSP-D) is produced in mammalian cells and bacteria (*Escherichia coli*), and also experimentally in the yeast *Pichia pastoris*. Mammalian cells produce full-size SP-D, but the yields are low and the cost of production is high. In contrast, bacteria produce a truncated form of SP-D, which is active *in vitro* and *in vivo*, and higher yields can be achieved at a lower cost. We compare the efficiency of production of rSP-D in terms of the total yields achieved in each system and the amount of SP-D needed to meet the global demand for the treatment of pulmonary diseases, using respiratory distress syndrome as a case study.

## Introduction

**Figure 1:**
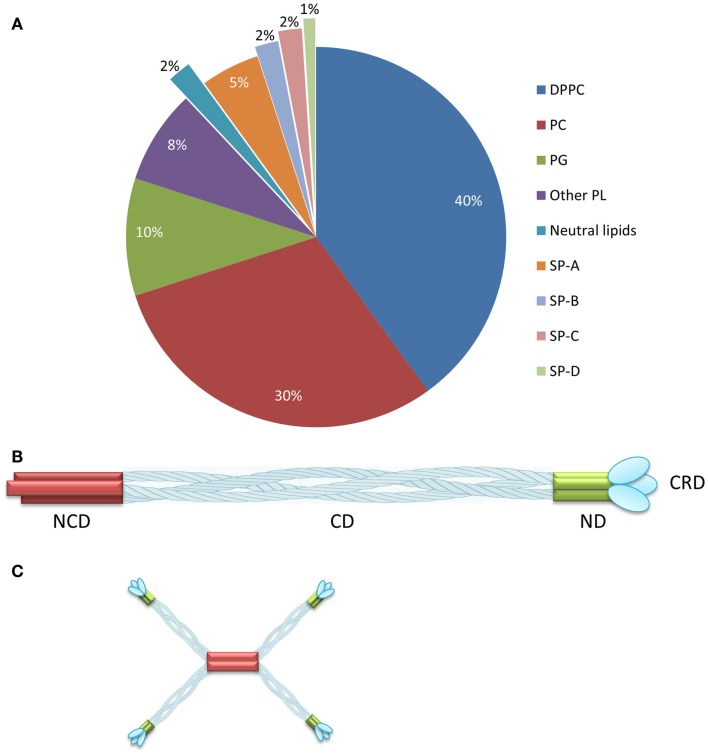
**Composition of pulmonary surfactant and SP-D oligomerization**. **(A)** Pulmonary surfactant is composed of lipids (90%) and proteins (10%) distributed as shown. **(B)** SP-D comprises four domains: the N-terminal, collagenous, neck, and carbohydrate-recognition domains. **(C)** SP-D assembles as a trimer, which forms higher multimeric forms such as dodecamers. Reproduced from Jobe and Ikegami (2) and Wright (7) with permission. DPPC, dipalmitoylphosphatidylcholine; PC, unsaturated phosphatidylcholine; PG, phosphatidylglycerol; PL, phospholipids; NCD, N-terminal non-collagenous domain; CD, collagenous domain; ND, α-helical-coiled coil neck domain; CRD, carbohydrate-recognition domain. **(A)** reproduced from Jobe and Ikegami (2), copyright (2001) with permission from Elsevier. **(B,C)** adapted from Wright (7), copyright (2005).

Mammalian pulmonary surfactant (PS) is a mixture of proteins (10%) and lipids (90%) including the major lipid component dipalmitoylphosphatidylcholine (DPPC) (1) (Figure 1A). Four classes of surfactant proteins are associated with surfactant lipids, named SP-A, SP-B, SP-C, and SP-D, representing 5, 2, 2, and 1% of the total PS composition by weight, respectively (2). The hydrophobic proteins SP-B and SP-C are necessary for the adsorption of the surfactant layer to the alveolar air–liquid interface, thus lowering the surface tension. The hydrophilic proteins SP-A and SP-D contribute to surfactant homeostasis and also play a role in innate immunity (3). The main function of the PS is to ensure minimal surface tension within the lung to avoid collapse during respiration. Furthermore, by interacting with inhaled pathogens, the PS also participates in host defense (4). PS deficiency is, therefore, associated with pulmonary diseases such as asthma, bronchiolitis, respiratory distress syndrome (RDS), cystic fibrosis, and pneumonia (5). A number of different exogenous surfactant preparations have been developed and tested in clinical trials (6). Curosurf^®^, a natural surfactant formulation derived from minced porcine lungs, is currently one of the leading surfactant products in USA, and comprises a mixture of phospholipids and the hydrophobic surfactant proteins SP-B and SP-C (6). Surfactant formulations are indicated for the treatment of RDS, which affects ~1.5 million premature babies globally every year (Box 1).

Box 1 **Calculating the annual demand for SP-D**

. To calculate the annual demand for SP-D, we used RDS as a case study because this is the only disease for which surfactant products are currently indicated. There are ~15 million premature births every year (8) and based on USA data only 10% of the premature babies are affected by RDS (9). Therefore, we used ~1.5 million babies as the basis for our annual demand calculations.

Surfactants are administered on the basis of body weight so to simplify the calculations we determined the mean body weight of a premature baby based on USA data from the Centers of Disease Prevention and Control (10). We defined a premature birth as any baby born weighing less than 2.5 kg and calculated the mean weight based on averaged frequency data [Table F in Ref. (10)] resulting in an average premature weight of 2 kg.

The recommended single dose of Curosurf^®^ per kg body weight (11) contains 2.5 mg of protein (SP-B plus SP-C), which represents a dose of 5 mg for a 2 kg premature baby. For more complicated cases, two additional doses are recommended within 72 h, so a three-dose regimen would administer 10 mg of protein, given that the second and third doses are half-strength. Because SP-B and SP-C together account for 40% of natural surfactant protein, whereas SP-D accounts for 10%, the corresponding “ideal” doses of SP-D to match the proportions present in natural surfactant (2) would be 1.25 mg for one dose and 2.5 mg for three doses. Multiplying these amounts by 1.5 million premature babies at risk of RDS we get minimum and maximum annual demands of 1.875 and 3.75 kg SP-D, respectively. For the truncated product [neck and carbohydrate-recognition domain (NCRD)], the same amount of functional protein would have 47% of the mass (12), so the minimum and maximum annual demands are reduced to 0.881 and 1.762 kg, respectively.

Respiratory distress syndrome is a major PS deficiency disease caused by the structural immaturity of the lungs in premature infants, which makes it difficult to breathe, inhibits gas exchange, and promotes alveolar collapse (13). However, treatment becomes more difficult if the lungs are infected or if there are inflammatory or oxidative complications, because current surfactant preparations lack SP-A and SP-D (1). The successful treatment of complex pulmonary diseases, therefore, requires the production of surfactant formulations whose composition matches natural PS as closely as possible (14).

Surfactant protein D has an important role in the pulmonary innate immune system by providing anti-inflammatory and antimicrobial activities that address chronic pulmonary diseases such as asthma, cystic fibrosis, and smoking-induced emphysema (15–18). Data based on premature newborn lambs suggest that the administration of ~2–3 mg/kg of recombinant human SP-D in combination with 100 mg/kg Survanta^®^ (a natural surfactant available in USA) is more effective than Survanta^®^ alone for the prevention of endotoxin shock and the reduction of lung inflammation caused by ventilation (19, 20).

Traditionally, SP-D has been isolated from the supernatant of bronchoalveolar lavage or amniotic fluid, but most SP-D is lost during the procedure because it is hydrophilic (21). The low SP-D yields and variable oligomerization states make it difficult to use natural sources for the production of pharmaceutical SP-D (22, 23). To overcome these limitations, recombinant SP-D (rSP-D) can be produced in microbes or mammalian cell lines, potentially offering a large-scale platform for the production of homogeneous rSP-D formulations. There is little data available concerning the global demand for rSP-D so we have used RDS as a case study, assuming that products such as Curosurf^®^ would benefit from the inclusion of rSP-D in the same proportion as found in natural PS. It would also be valuable to gain insight into the productivity of different rSP-D production platforms, comparing their advantages and disadvantages to develop an effective production strategy.

## Surfactant Products in Clinical Use

The exogenous surfactants tested in RDS clinical trials can be assigned to three groups. The first comprises modified natural surfactants of bovine or porcine origin, which contain a mixture of phospholipids but only the hydrophobic proteins SP-B and SP-C, e.g., Curosurf^®^, BLES^®^, Infasurf^®^, and Survanta^®^ (6). The second comprises the synthetic surfactants Surfaxin^®^ and Venticute^®^. The former contains DPPC, phosphatidylglycerol (PG), palmitic acid, and a protein analog KL-4 (sinalputide), which mimics the activity of SP-B (1, 24). The latter contains DPPC, PG, palmitic acid, and recombinant human SP-C (1, 25). Finally, the third group comprises protein-free synthetic surfactants featuring a mix of phospholipids and additives (e.g., ALEC^®^ and Exosurf^®^) (1). Clinical trials have been carried out to compare approved surfactant products for the treatment of neonates with RDS. The use of synthetic surfactants, which were initially promoted as a less expensive product with homogeneous composition and a low risk of contamination with animal pathogens, has declined due to their poor clinical performance and complex manufacturing process (1, 26). Synthetic surfactants should contain at least one hydrophobic protein or analog for optimal results, but these are structurally complex or unstable in pure form (27). Although modified natural surfactants are more expensive (~$500 per dose for neonates), they also reduce mortality and pulmonary air leaks more successfully (13, 28). Nevertheless, there is a higher risk of contamination with pathogens when animal-derived products are used, and the modified natural surfactants have a low and variable protein content compared to natural surfactants. For example, Survanta^®^ contains only ~12% of the SP-B content and ~50% of the SP-C content compared to the endogenous bovine surfactant, whereas Curosurf^®^ contains only ~33% of the SP-B content and ~50% of the SP-C content compared to the endogenous porcine surfactant (1, 29).

The *in vitro* activity of animal-derived surfactants shows variable sensitivity to inhibition by plasma proteins, fatty acids, and proteases that eventually inactivate endogenous PS, based on the different protein contents of these products, making them more resistant when the surfactant proteins are present in greater amounts (1, 30). The concentration of SP-B and SP-C in surfactant products must ensure the efficient adsorption and spreading of phospholipids. Curosurf^®^ is the most widely used product for the treatment of RDS (31). This natural surfactant contains 1 mg/ml of SP-B and SP-C proteins (11, 32) and one dose of 2.5 ml/kg body weight is recommended followed if necessary by second and third doses of 1.25 ml/kg each (11). Assuming an average premature birth weight of 2 kg (Box 1), this means the average dose of SP-B plus SP-C is 5 mg for one treatment and 10 mg for three treatments. With 1.5 million premature babies affected by RDS every year, this equates to a global demand of between 7.5 and 15 kg of SP-B plus SP-C to ensure enough supplies for each child to receive one or three doses, as best and worst case scenarios.

## Structure and Functions of SP-D

Surfactant protein D is a glycoprotein that belongs to the family of collagenous carbohydrate-binding proteins known as collectins (33–35). This group includes SP-A, serum mannose-binding protein (MBL), conglutinin, and CL-43. Collectins comprise four domains: a cysteine-linked N-terminal region required for the formation of intermolecular disulfide bonds, a triple-helical collagen region, an α-helical-coiled-coil trimerizing neck peptide, and a C-terminal calcium-dependent carbohydrate-recognition domain (CRD) (36) (Figure 1B). SP-D is assembled as trimer (129 kDa in total, comprising three identical 43-kDa polypeptide chains), but higher oligomerization states such as dodecamers can also be formed (36, 37) (Figure 1C). SP-D is an innate host defense molecule that interacts directly with carbohydrates on the surface of pathogens including bacteria, viruses, fungi, and protozoa. These interactions cause pathogen aggregation followed by the activation of phagocytes to destroy them (37, 38). A higher degree of SP-D oligomerization increases the recognition and binding of carbohydrate ligands to the pathogen surface (37).

## Natural Sources of SP-D

The structure of SP-D from human, murine, porcine, and bovine sources has been studied to determine its function in the innate immune system (39–43). SP-D is usually isolated from bronchoalveolar lavage during alveolar proteinosis (the abnormal accumulation of surfactant in the alveoli, interfering with gas exchange) followed by carbohydrate affinity chromatography (21, 23).

The use of natural SP-D to supplement PS formulations is the best option to ensure therapeutic efficiency because higher-order multimerization in the endogenous surfactant increases the number of SP-D-binding sites to carbohydrate ligands on the surface of pathogens, achieving potent bacterial and viral agglutination effects (44). However, the SP-D concentration after lung lavage is low because the hydrophilic properties of SP-D cause most of the protein to be lost during extraction (45). Animal sources also present a risk of contamination with pathogens as well as non-uniform SP-D composition, reflecting the different oligomerization states that form after extraction and purification (22, 23).

## Heterologous SP-D Production Systems

### Mammalian cell lines

One of the first *in vivo* assays using prematurely delivered lambs demonstrated the positive effects of Survanta^®^, a natural commercial surfactant, supplemented with full-size rSP-D produced by Chinese hamster ovary (CHO) cells. A dose of 2 mg/kg recombinant human SP-D improved the surfactant function by protecting the premature lung against inflammation induced by ventilation. This study was one of the first to indicate the benefits of adding a full-size rSP-D to the natural surfactant product and its potential use for the treatment of pulmonary diseases (19, 20, 46).

The production of active therapeutic proteins depends not only on protein synthesis but also correct folding and post-translational modification, especially glycosylation (47). SP-D folds with the aid of disulfide bonds in the N-terminal region and the collagen region also undergoes N-glycosylation (37). Therefore, SP-D is usually synthesized in mammalian cells because they produce authentic glycan structures (43). Despite the typical advantages of mammalian cells in terms of yields and post-translation modifications (48, 49), the production of rSP-D remains a challenge because it is not synthesized efficiently. The mammalian cell line that is most widely used for the production of full-length SP-D is the CHO-K1 subclone (48). CHO cells can produce many biopharmaceutical products in the grams per liter yield range following extensive cell line and process optimization (50), but in the case of rSP-D, the yields are typically 0.5–2.0 mg of purified protein per liter (51). If we match the demand for rSP-D against the current annual use of Curosurf^®^ for the treatment of RDS (34), it would be necessary to produce 1.875–3.75 kg/year based on a single dose for the minimum demand and three doses for the maximum demand, in each case representing ~1.5 million premature babies (Box 1). Even if rSP-D could be produced by industrial fermentation in 20,000-l bioreactors (52, 53), each campaign would only produce a maximum of 40 g of purified protein per campaign, so even with 100% success at the highest current yields this would require 47–94 campaigns per year to meet the annual demand for this protein.

Human embryonic kidney cell line 293 (HEK293) has also been used to produce rSP-D and in this case yields were reported in the range 1–5 mg/l (54). Using the same assumptions as above, this suggests that a single campaign in a 20,000-l fermenter would yield 100 g of pure rSP-D, requiring 19–38 campaigns to meet annual demand (52, 53).

As well as the large volumes of mammalian cell culture required to produce sufficient amounts of rSP-D to meet global demand, such cell lines also present an additional risk of contamination with animal pathogens, which increases the costs of downstream processing and purification, and hence the cost of the production facilities (55). However, mammalian cells are advantageous over natural sources of SP-D because they provide continuous and uniform amounts of protein over a short cultivation period and the source material is not scarce. SP-D yields could be improved in the future by medium optimization, the selection of better production cell lines and the optimization of cultivation strategies (55).

#### Escherichia coli

*Escherichia coli* was the first organism used for the production of therapeutic recombinant proteins and is still widely used today, particularly for the production of small proteins lacking glycan structures. Cultivation is simple and inexpensive, and large amounts of protein can be produced in a relatively short time (55). *E. coli* has been used successfully for the production of a truncated form of rSP-D (35, 56–58) comprising only the NCRD. These components of the protein are not glycosylated and do not require any other post-translational modifications, but retain the biological activity of the full-size protein *in vitro* and *in vivo* (57) because they undergo normal CRD folding, intramolecular disulfide bond formation, the co-ordination of calcium ions, and ligand binding (12, 58–60). Human, rat, and mouse versions of NCRD have been produced successfully in *E. coli* (58). The yields of purified mouse NCRD (5–10 mg/l) were about four times lower than human and rat NCRD, suggesting that the maximum yield of human NCRD is 40 mg/l in this system. The Arg-Ala-Lys (RAK) sequence from CL-43 bovine serum collectin was inserted into the corresponding SP-D sequence and this modified protein was also expressed with a yield approaching 40 mg/l (56). Trimeric NCRD is a 60 kDa polypeptide but each molecule has the same activity as the trimeric full-size rSP-D, which is 129 kDa (12). This means that every kg of the full-size product can be replaced with 470 g of the truncated derivative. On this basis, the annual demand for active rSP-D can be met by producing 0.881–1.762 kg of NCRD (Box 1). Again assuming a campaign based on a 20,000-l bioreactor, the entire annual demand for NCRD could be met by 1–2 campaigns (52, 53).

Several *in vivo* studies have demonstrated the therapeutic effects of purified recombinant NCRD produced in *E. coli* in mouse models of infectious, allergic, and inflammatory diseases. The administration of recombinant NCRD suppressed the development of allergy symptoms against *Aspergillus fumigatus* (12, 61) and *Dermatophagoides pteronyssinus* (18, 62). In addition, the intrapulmonary administration of recombinant NCRD also reduced the number of apoptotic and necrotic alveolar macrophages, helped to control asthma-related inflammation and improved lung health in SP-D-deficient mice (63).

The successful preclinical testing of recombinant NCRD produced in *E. coli* demonstrates the suitability of this platform for the production of an active pharmaceutical ingredient for human use. The yields can be improved by optimizing gene expression and protein accumulation (e.g., by using different promoters to boost gene expression, incorporating a leader peptide to direct the protein into the periplasmic space or by expressing fusion proteins to increase product stability) and by improving the growth medium and process parameters (55).

#### Pichia pastoris

Yeast provide cost–effective production systems with high productivity and rapid growth like bacteria, but they are eukaryotic cells and can, therefore, fold complex proteins and carry out most forms of post-translational modification (55). Despite these advantages, rSP-D is not yet produced in yeast, although *Pichia pastoris* has been used to produce truncated SP-D to enable the analysis of its crystal structure. The yield of human NCRD in *P. pastoris* was 7 mg/l (60), which means that 140 g of purified NCRD could be produced by *P. pastoris* cells using a 20,000-l fermenter (52, 53). Based on the calculations presented above, this would require 6–13 campaigns to meet annual demand. The capacities of the production platforms compared in this review are summarized in Table 1.

**Table 1:** **The ability of heterologous production systems to meet the current global demand for recombinant SP-D**.

Heterologous production system	Maximum reported yields (mg/l)	Number of campaigns required to meet minimum annual demand^a^	Number of campaigns required to meet maximum annual demand^b^
**FULL-SIZE SP-D**			
CHO-K1	2	47	94
HEK293	5	19	38
**NCRD**			
*Escherichia coli*	40	1	2
*Pichia pastoris*	7	6	13

*^a^Minimum annual demand is 1.875 kg rSP-D or 0.881 kg NCRD (see Box 1)*.

*^b^Maximum annual demand is 3.75 kg rSP-D or 1.762 kg NCRD (see Box 1)*.

*The reported maximum yields of purified full-size recombinant SP-D and its truncated form (NCRD) in different heterologous production systems were used to estimate the number of campaigns required to meet annual demand assuming ~1.5 million premature babies require one treatment per year (minimum demand) or three treatments per year (maximum demand) and that each campaign has a production volume of 20,000 l. The calculations are explained in detail in Box 1*.

### Future perspectives

In addition to the heterologous expression systems discussed above, alternative platforms can be developed to make the production of rSP-D more efficient. For example, insect cells carry out more complex post-translational modifications than either *E. coli* or *P. pastoris*, and they also have the ability to fold and assemble complex proteins, which should enhance the formation of SP-D dodecamers and higher order multimers (55). SP-A and SP-B have already been produced using insect cells, thus demonstrating the capacity of this system to produce complex surfactant proteins (64, 65). However, insect cells require more expensive media than bacteria, which makes the production of large quantities of protein more cost intensive (66).

Surfactant protein D could also be produced in transgenic plants or plant cell suspension cultures, which offer economic benefits for the large-scale of production of pharmaceutical proteins (67, 68). Upstream production in plants is less expensive than all other systems because only light, water, and basic nutrients are needed for growth, and cultivation can be implemented on an agricultural scale without bioreactors or skilled labor (55). However, downstream processing is more expensive compared to mammalian or microbial culture media and represents up to 80% of the total costs of therapeutic protein production (69–71). The development of new purification strategies would, therefore, be necessary for the cost–effective production of SP-D in plants.

During the last 5 years, the structural characteristics of SP-D have been solved in detail and this knowledge now makes it possible to design synthetic peptide analogs, a new production strategy that could replace native and rSP-D in future artificial surfactants. For example, Surfaxin^®^ contains sinalputide (KL_4_ peptide), a poly-N-substituted glycine analog that mimics the function of SP-B, and this promotes healthy surfactant film morphology and adsorption (72). However, SP-D is significantly larger than SP-B and is organized as a trimeric structure comprising several amphipathic helices, which can assemble into dodecameric or higher multimeric complexes. Therefore, the development of SP-D analogs is likely to be more complex. It is possible that small peptide analogs could be developed to mimic the functions of the neck domain together with the CRD as an alternative to rSP-D and NCRD.

## Conclusion

Heterologous expression platforms are used for the production of SP-D because only small amounts of the protein can be isolated from animals, and this is insufficient to meet the global demand. Surfactants are widely used for the treatment of RDS and would benefit from the inclusion of rSP-D, which has shown promising results in clinical trials for infectious and inflammatory lung diseases. The global demand in the context of RDS is currently in the low kilogram per year range and can be met by weekly campaigns in mammalian cells, which can produce full-size SP-D. The complete SP-D molecule is essential for innate defense against acute bacterial or viral respiratory infections because it achieves high-affinity binding to lipopolysaccharide and causes the subsequent agglutination of the pathogens. However, this platform can be susceptible to contamination with animal pathogens, which limit its production in mammalian systems. As an alternative, the global demand for rSP-D in the context of RDS can be met with only 1–2 large-scale campaigns in *E. coli* for the production of a truncated fragment of SP-D lacking the collagenous domain. The truncated version of the protein can reduce the total number of apoptotic macrophages during chronic lung inflammation in mice deficient for SP-D. The demand for SP-D will grow when it is approved for other indications such as asthma and cystic fibrosis, and current systems will not produce sufficient quantities of the protein. Further research is therefore required to develop efficient production platforms based on a wider range of expression systems including yeast, insect cells, and plants, to ensure that the production capacity for SP-D meets the growing demand for this protein.

## Author Contributions

All authors contributed equally to the analysis and interpretation of the data and to the preparation of the manuscript.

## Conflict of Interest Statement

The authors declare that the research was conducted in the absence of any commercial or financial relationships that could be construed as a potential conflict of interest.
